# Examining Caregiver Burden in Low- and Middle-Income Countries: A Scoping Review

**DOI:** 10.1093/geroni/igaf122.3760

**Published:** 2025-12-31

**Authors:** Wenjie Chen, Aasha Paredes, Yiwei Zhang, Christian Noval, Wai Yan Min Htike, Haolin Li, Chenkai Wu, Hanzhang Xu

**Affiliations:** Duke Kunshan University, Kunshan, Jiangsu, China (People’s Republic); Duke University, Durham, North Carolina, United States; Duke Kunshan University, Suzhou, Jiangsu, China (People’s Republic); Duke University, Durham, North Carolina, United States; Duke Kunshan University, Kunshan, Jiangsu, China (People’s Republic); Duke Kunshan University, Kunshan, Jiangsu, China (People’s Republic); Duke Kunshan University, Kunshan, Jiangsu, China (People’s Republic); Duke University, Durham, North Carolina, United States

## Abstract

Most research regarding caregiver burden was conducted in high-income countries. However, for low- and middle-income countries (LMICs), structural challenges and limited resources exacerbate caregiver burden. For instance, while government support programs and formal caregivers exist, they are largely inaccessible and unaffordable. Additionally, long-term care systems are underdeveloped overall leaving family members as the primary source of care. This scoping review aims to describe current evidence on caregiver burden in LMICs, including the severity and types of caregiver burden, factors associated with caregiving burden, and existing interventions. We applied PRISMA extension for scoping reviews and searched PubMed for relevant articles up to Aug 2024. We used the Joanna Briggs Institute Manual for Evidence Synthesis and Critical Appraisal Checklist to evaluate quality of evidence. A total of 43 peer-reviewed articles were included, encompassing data from 15,503 caregivers across 12 countries, spanning Asia, Latin America, and Africa. On average, caregivers reported moderate to high levels of perceived burden, particularly in emotional, physical, and social domains. Higher caregiver burden was observed among older, female caregivers characterized by lower educational attainment and limited social support. The most frequently examined care recipient factors included cognitive impairment and depressive symptoms. Common interventions to reduce caregiver burden include structured caregiving activities, discharge planning, spiritual therapy, and social-emotional support. Findings from this scoping review highlight substantial emotional, physical, and social burden faced by caregivers in LMICs, particularly among women. We also found considerable variability in the measures of caregiving burden, study design, and quality of the evidence across studies.

